# MicroRNA profiles in preterm children with regard to BPD and echocardiographic findings

**DOI:** 10.3389/fmed.2026.1913675

**Published:** 2026-08-04

**Authors:** Ilona Hromadnikova, Katerina Kotlabova, Ivan Berka, Jan Sirc

**Affiliations:** 1 Department of Molecular Biology and Cell Pathology, Third Faculty of Medicine, Gynaecology and Obstetrics Clinic, Institute for the Care of Mother and Child, Charles University, Prague, Czechia; 2 Third Faculty of Medicine, Neonatology Clinic, Institute for the Care of Mother and Child, Charles University, Prague, Czechia

**Keywords:** bronchopulmonary dysplasia, echocardiographic examination, microRNA, prematurity, preschool/school-age children, pulmonary vascular resistance

## Abstract

**Background:**

The associations between microRNA profiles in peripheral blood leukocytes and previous occurrence of bronchopulmonary dysplasia (BPD) and current echocardiographic findings were studied in preschool and school-age preterm children with a birth weight < 1,500 grams and/or gestational age at birth < 32 weeks.

**Methods:**

The prospective study was conducted in 152 children with a median age of 5.42 years diagnosed (*n* = 46) or not diagnosed (*n* = 106) with BPD and 92 age-matched normal term children (controls).

**Results:**

A higher incidence of abnormal echocardiographic findings was detected in preterm children regardless of BPD occurrence compared to normal term controls. Preterm children shared altered microRNA profiles regardless of BPD occurrence (20 microRNAs in total; 17 upregulated, 3 downregulated). Additional 3 microRNAs were dysregulated in children without BPD and 1 microRNA in children with BPD. MiR-199a-5p differentiated between children with and without BPD. No significant differences in microRNA expression profiles were observed either among normal term controls or within the cohort of preterm children when stratified according to echocardiographic findings.

**Conclusion:**

Preterm children of preschool and school age show a higher incidence of abnormal echocardiographic findings compared to term-born controls. Although no association was identified between these findings and microRNA expression profiles, the impact of prematurity on microRNA expression appears to be well-defined. These results may contribute to the growing body of evidence emphasizing the importance of long-term follow-up in individuals born preterm, while further research is warranted to determine whether the observed microRNA alterations are predictive of later health complications.

## 1 Introduction

Bronchopulmonary dysplasia (BPD) is a multifactorial chronic lung disease characterized by disrupted alveolar and vascular development, which occurs most commonly (up to 40–45%) in infants with extremely low birth weight (ELBW) or birth before 29 gestational weeks (1–3).

The disease may develop due to other adverse factors, especially under the influence of mechanical ventilation, oxygen toxicity, postnatal inflammation and hospital-acquired infections, fetal growth restriction, nutritional deficits and genetic predisposition (2, 4, 5). In severe cases, the development of secondary pulmonary hypertension (PH) has been observed (6).

The definition of BPD has been revised several times over the years (7–14). The current definition is based on radiographic confirmation of parenchymal lung disease, the need to use supplemental oxygen treatment for **≥** 3 consecutive days to maintain arterial oxygen saturation in the 90–95% range or oxygen and respiratory support at 36 weeks postmenstrual age (PMA) for infants less than 32 weeks gestational age (15). Furthermore, the current most widely accepted definition divides BPD according to severity of disease (grades I, II and III), with grade III corresponding to the most severe form (15). The Executive Summary of a Workshop on BPD also highlighted the need to obtain data on the short- and long-term outcomes of BPD to set clinical recommendations for improving respiratory and other outcomes of preterm infants (15). Respiratory distress syndrome (RDS) was defined as progressive increased work of breathing and the need for oxygen therapy and/or respiratory support in the first 6 h of life.

Severe respiratory morbidity (hospitalization for respiratory reasons at ≥ 45 PMA weeks, oxygen therapy or respiratory monitoring at discharge, or rehospitalization for respiratory diseases (acute bronchitis or pneumonia ≥ 2 times) was demonstrated in children born before 32 weeks diagnosed with BPD at the corrected age of 18–24 months. The incidence of adverse outcomes (late death or severe respiratory morbidity) increased with BPD severity from 28.8% for grade I to 71.8% for grade III (16).

Individuals with BPD have been observed to have a higher incidence of respiratory problems requiring hospitalization and persistent lung function abnormalities leading to the development of small airway disease and asthma-like symptoms in school-age children and the early development of chronic obstructive pulmonary disease (COPD) and PH (17–19). However, some studies show no association between BPD and respiratory dysfunction in preschool or school-age children born with extremely low birth weight. Respiratory infections, asthma and increased airway hyperresponsiveness were generally more common in extremely preterm infants (20–22). According to a meta-analysis, an increasing incidence of PH was observed with advanced BPD severity (5, 18, and 41% for mild, moderate and severe BPD, respectively) (23). Next, impaired neurodevelopment and an increased incidence of cerebral palsy were demonstrated in individuals diagnosed with BPD (17, 18, 24).

Extremely preterm birth is associated with long-term alterations in cardiac structure and/or function, particularly in individuals diagnosed with BPD (25–28). Echocardiographic studies have revealed that, compared with term-born children, ex-preterm preschool or school children often present with smaller left and/or right ventricular dimensions (25, 26) and subclinical impairments in left ventricular systolic and diastolic function (25). Right heart and pulmonary vascular changes are also prominent, including increased relative wall thickness, higher estimated pulmonary vascular resistance (PVR), and reduced right ventricular systolic performance (26). Children with BPD further demonstrate significantly lower tricuspid annular plane systolic excursion (TAPSE) and TAPSE/PASP ratios, indicating compromised right ventricular function despite the absence of overt PH (25). Additionally, neonatal presence of a patent ductus arteriosus (PDA) has been linked to higher PVR and right ventricular myocardial performance index (RVmpi) in ex-preterm children, although no consistent association with BPD has been found (26). Echocardiographic findings further support the presence of pulmonary and cardiac sequelae in this population. Compared with preterm born children with mild or no BPD school-age children born preterm with moderate-to-severe BPD exhibit a higher prevalence of an R/S wave ratio > 1 in lead V1, typically associated with elevated pulmonary artery pressure, and more vertical P wave axes, usually associated with emphysematous changes (27). Importantly, Datora et al. (28) demonstrated that some of these cardiovascular alterations persist into young adulthood. Extremely preterm born young adults (aged 18–29 years), particularly those with a history of BPD, continue to show reduced right ventricular systolic function (28). These findings underscore the need for long-term cardiovascular monitoring in individuals born extremely preterm to better understand the progression and clinical implications of these phenotypes (25–28).

The objectives of the study were to evaluate the incidence of abnormal echocardiographic findings in preschool and school-age preterm children with a birth weight < 1,500 grams and/or gestational age at birth < 32 weeks and to compare them with age-matched normal term controls. Additionally, we analyzed microRNA expression profiles in venous peripheral blood leukocytes of children, investigating their association with prior BPD occurrence and current abnormal echocardiographic findings, with the objective of identifying potential biomarkers predictive of increased PVR as a risk factor for late-onset PH following BPD.

## 2 Materials and methods

### 2.1 Patients cohort

The study was conducted in preterm children aged 4.17–9.50 years (median 5.42 years). Inclusion criteria were birth weight < 1,500 grams and/or gestational age at birth < 32 weeks. All experiments were approved by the local ethical committees (the Ethics Committee of the Third Faculty of Medicine, Charles University and the Ethics Committee of the Institute for the Care of the Mother and Child, Charles University). All patients were enrolled after prior parental agreement, and parents also provided written informed consent to participate in the study. Study protocol was conducted following the standards of the Declaration of Helsinki.

**FIGURE 1:**
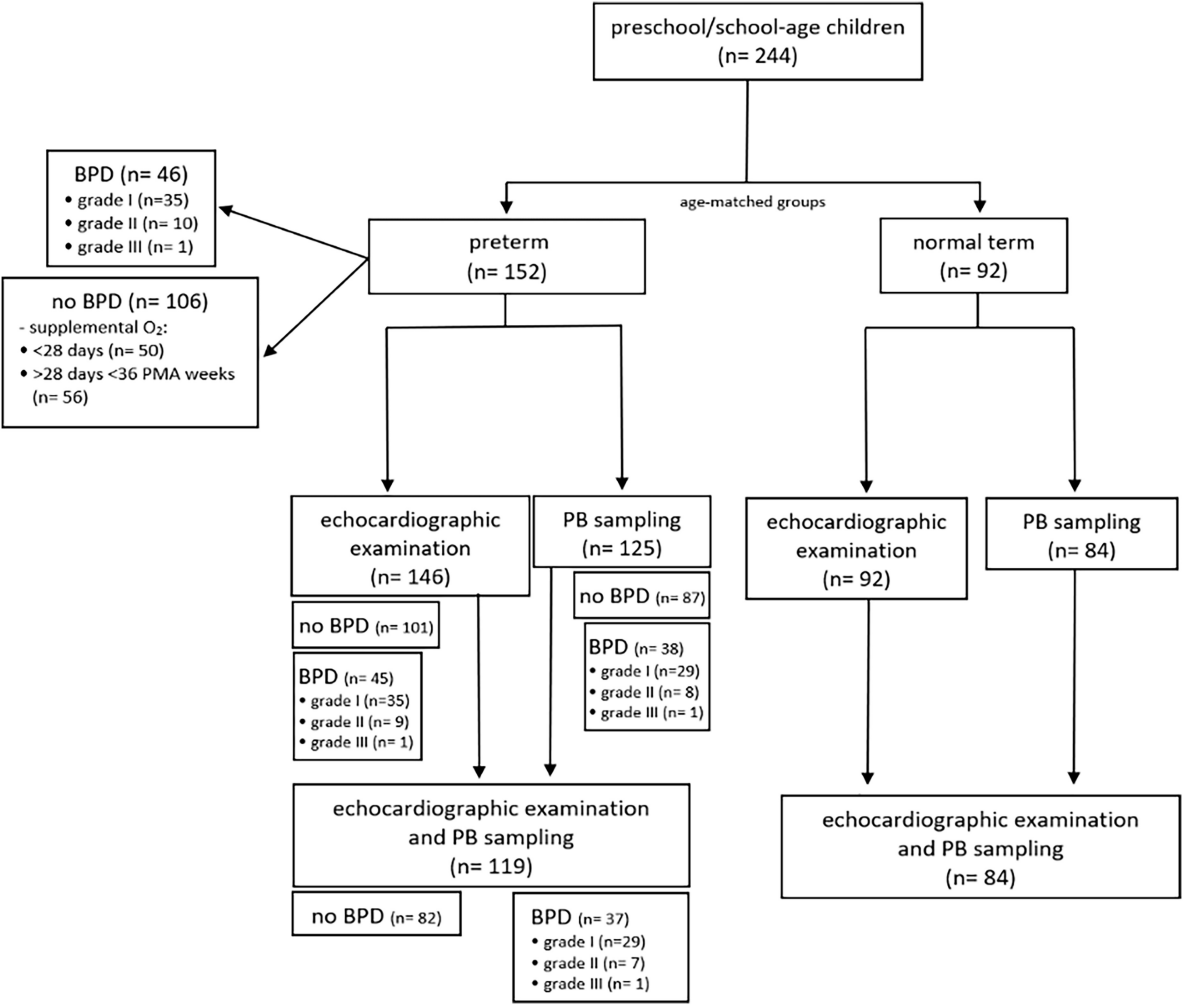
Flow chart of the participants included in the study. The flow chart of detailed specifications of study participants summarizes the number of children in each group based on which examinations, or combinations thereof, they were able to complete. BPD, bronchopulmonary dysplasia; O_2_, oxygen; PMA, postmenstrual age; PB, peripheral blood.

A total of 152 preterm children were included in the study, of whom 46 were diagnosed with BPD. The control group consisted of 92 age-matched normal term children (median 5.67 years, 3.83–11.17 years). All participants were invited to undergo echocardiographic examination and peripheral blood sampling. However, not all children were able to complete both procedures due to distress or other limiting factors. The final number of children in each group, based on which examinations or combinations thereof they were able to complete, is presented in Figure 1. Baseline clinical characteristics are summarized in Table 1.

**TABLE 1:** Baseline characteristics of cases and controls.

Baseline characteristics		Normal term (*n* = 84)	No BPD preterm (*n* = 87)	BPD preterm (*n* = 38)	*p*-value^1^ OR 95% CI	*p*-value^2^ OR 95% CI	*p*-value^3^ OR 95% CI
At follow up							
Age (years)		5.67 (3.83–11.17)	5.50 (4.58–9.17)	5.33 (4.17–8.83)	0.564	0.445	0.077
Height (cm)		118 (98–153)	114 (100–139)	113.5 (99–139)	0.792	0.064	0.066
Weight (kg)		21.5 (14–40.8)	20.0 (13.6–45.0)	18.65 (12.5–35.0)	0.166	0.003	0.051
BMI (kg/m^2^)		15.43 (13.22–20.0)	14.6 (12.02–23.63)	14.05 (11.34–21.36)	0.001	< 0.001	0.295
Asthma diagnosis		0 (0%)	3 (3.45%)	2 (5.26%)	*p* = 0.200 OR = 7.000 (0.356–137.614)	*p* = 0.117 OR = 11.575 (0.542–247.154)	*p* = 0.636 OR = 1.556 (0.249–9.710)
Allergy		5 (5.95%)	17 (19.54%)	7 (18.42%)	*p* = 0.012 OR = 3.837 (1.346–10.941)	*p* = 0.041 OR = 3.568 (1.053–12.091)	*p* = 0.884 OR = 0.930 (0.350–2.469)
Chronic respiratory infections		4 (4.76%)	12 (13.79%)	4 (10.53%)	*p* = 0.052 OR = 3.200 (0.989–10.358)	*p* = 0.245 OR = 2.353 (0.556–9.959)	*p* = 0.616 OR = 0.735 (0.221–2.446)
Gestation/Maternal data							
Maternal age at delivery (years)		32 (21–46)	32 (22–48)	32.5 (22–49)	0.684	0.466	0.694
Infertility treatment	Yes No	2 (2.38%) 82 (97.62%)	21 (24.14%) 66 (75.86%)	9 (23.68%) 29 (76.32%)	*p* < 0.001 OR = 13.046 (2.951–57.664)	*p* = 0.002 OR = 12.724 (2.596–62.374)	*p* = 0.956 OR = 0.975 (0.399–2.386)
Mode of delivery	Vaginal CS	76 (90.48%) 8 (9.52%)	15 (17.24%) 72 (82.76%)	4 (10.53%) 34 (89.47%)	< 0.001	<0.001	0.336
Twin/multiple birth		0 (0%)	41 (47.13%)	21 (55.26%)	*p* < 0.001 OR = 150.828 (9.068–2508.695)	*p* <0.001 OR = 207.629 (12.010–3592.185)	*p* = 0.403 OR = 1.386 (0.645–2.980)
Antenatal steroids	No Partial Completed	84 (100.0%) 0 (0%) 0 (0%)	5 (5.75%) 18 (20.69%) 64 (73.56%)	2 (5.26%) 8 (21.05%) 28 (73.68%)	ND	ND	0.994
MgSO_4_		0 (0%)	26 (29.89%)	14 (36.84%)	*p* = 0.003 OR = 72.821 (4.353–1218.181)	*p* = 0.002 OR = 100.020 (5.757–1737.608)	*p* = 0.444 OR = 1.369 (0.613–3.056)
Antenatal antibiotics		14 (16.67%)	44 (50.57%)	23 (60.53%)	*p* < 0.001 OR = 5.116 (2.512–10.420)	*p* <0.001 OR = 7.667 (3.220–18.252)	*p* = 0.306 OR = 1.499 (0.691–3.251)
Preeclampsia		0 (0%)	14 (16.09%)	6 (15.79%)	*p* = 0.015 OR = 33.340 (1.955–568.662)	*p* = 0.018 OR = 33.800 (1.851–617.244)	*p* = 0.966 OR = 0.978 (0.345–2.774)
Gestational hypertension		0 (0%)	2 (2.30%)	1 (2.63%)	*p* = 0.305 OR = 4.942 (0.234–104.474)	*p* = 0.245 OR = 6.760 (0.269–169.815)	*p* = 0.911 OR = 1.149 (0.101–13.065)
GDM		0 (0%)	6 (6.90%)	3 (7.89%)	*p* = 0.078 OR = 13.479 (0.747–243.141)	*p* = 0.065 OR = 16.662 (0.839–331.023)	*p* = 0.843 OR = 1.157 (0.274–4.891)
PPROM		0 (0%)	21 (24.14%)	8 (21.05%)	*p* = 0.006 OR = 54.639 (3.250–918.739)	*p* = 0.009 OR = 47.098 (2.638–840.797)	*p* = 0.707 OR = 0.838 (0.334–2.106)
Chorioamnionitis		0 (0%)	20 (22.99%)	6 (15.79%)	*p* = 0.006 OR = 51.326 (3.048–864.232)	*p* = 0.018 OR = 33.800 (1.851–617.244)	*p* = 0.364 OR = 0.628 (0.230–1.716)
Maternal smoking (during pregnancy)		1 (1.19%)	1 (1.15%)	1 (2.63%)	*p* = 0.980 OR = 0.965 (0.059–15.685)	*p* = 0.572 OR = 2.243 (0.137–36.845)	*p* = 0.555 OR = 2.324 (0.142–38.166)
Neonatal characterictics							
Gestational age (weeks)		39.86 (37.71–41.86)	29.29 (24.0–31.71)	26.22 (23.71–30.86)	p < 0.001	<0.001	< 0.001
Gestational age < 28 weeks (Extremely preterm children)		0 (0%)	20 (22.99%)	27 (71.05%) 11 (28.95%)	*p* = 0.007 OR = 48.896 (2.903–823.651)	*p* < 0.001 OR = 385.000 (21.952–6752.193)	*p* <0.001 OR = 8.223 (3.477–19.447)
Gestational age ≥ 28 - < 32 weeks (Very preterm children)		0 (0%)	67 (77.01%)				
Birth weight (g)		3395 (2530–4450)	1075 (540–1490)	745 (322-1490)	< 0.001	<0.001	< 0.001
Sex	Boy Girl	44 (52.38%) 40 (47.62%)	54 (62.07%) 33 (37.93%)	26 (68.42%) 12 (31.58%)	0.200	0.097	0.496
FGR		0 (0%)	12 (13.79%)	8 (21.05%)	*p* < 0.022 OR = 27.980 (1.629–480.697)	*p* < 0.009 OR = 47.098 (2.638–840.797)	*p* < 0.312 OR = 1.667 (0.619–4.484)
SGA		0 (0%)	8 (9.20%)	1 (2.63%)	*p* < 0.048 OR = 18.069 (1.026–318.245)	*p* < 0.245 OR = 6.760 (0.269–169.815)	*p* < 0.221 OR = 0.267 (0.032–2.213)
Apgar score < 7 at 1 min		0 (0%)	53 (60.92%)	33 (86.84%)	*p* < 0.001 OR = 262.073 (15.734–4365.188)	*p* <0.001 OR = 1029.364 (55.371–19136.133)	*p* < 0.006 OR = 4.234 (1.505–11.913)
Apgar score < 7 at 5 min		0 (0%)	9 (10.34%)	10 (26.32%)	*p* < 0.039 OR = 20.452 (1.171–357.276)	*p* < 0.005 OR = 62.263 (3.535–1096.687)	*p* < 0.027 OR = 3.095 (1.140–8.403)
RDS		0 (0%)	83 (95.40%)	38 (100.0%)	*p* < 0.001 OR = 3135.889 (166.212–59164.369)	*p* <0.001 OR = 13013.000 (253.464–668095.666)	*p* = 0.344 OR = 4.150 (0.218–79.018)
Pulmonary hemorrhage		0 (0%)	2 (2.30%)	6 (15.79%)	*p* = 0.305 OR = 4.942 (0.234–104.474)	*p* = 0.018 OR = 33.800 (1.851–617.244)	*p* = 0 014 OR = 7.969 (1.529–41.541)
Late-onset sepsis		0 (0%)	14 (16.09%)	16 (42.11%)	*p* = 0.015 OR = 33.340 (1.955–568.662)	*p* < 0.001 OR = 123.933 (7.157–2146.190)	*p* = 0.002 OR = 3.792 (1.603–8.973)
PDA		0 (0%)	21 (24.14%)	26 (68.42%)	*p* = 0.006 OR = 54.639 (3.250–918.739)	*p* < 0.001 OR = 358.280 (20.513–6257.600)	*p* < 0.001 OR = 6.810 (2.934–15.806)
NEC		0 (0%)	1 (1.15%)	3 (7.89%)	*p* = 0.512 OR = 2.931 (0.118–72.959)	*p* = 0.065 OR = 16.662 (0.839–331.023)	*p* = 0.088 OR = 7.371 (0.741–73.309)
PIVH		0 (0%)	6 (6.90%)	1 (2.63%)	*p* = 0.078 OR = 13.479 (0.747–243.141)	*p* = 0.245 OR = 6.760 (0.269–169.815)	*p* = 0.359 OR = 0.365 (0.042–3.140)
PVL		0 (0%)	3 (3.45%)	0 (0%)	*p* = 0.200 OR = 7.000 (0.356–137.614)	*p* = 0.696 OR = 2.195 (0.043–112.683)	*p* = 0.447 OR = 0.314 (0.016–6.220)
ROP (grade 3, 4)		0 (0%)	1 (1.15%)	14 (36.84%)	*p* = 0.512 OR = 2.931 (0.118–72.959)	*p* = 0.002 OR = 100.020 (5.757–1737.608)	=*p* < 0.001 OR = 50.167 (6.276–401.011)
Delivery room interventions							
Oxygen		0 (0%)	81 (93.10%)	37 (97.37%)	*p* < 0.001 OR = 2119.000 (117.467–38224.967)	*p* < 0.001 OR = 4.225 (168.189–106134.638)	*p* = 0.359 OR = 2.741 (0.319–23.587)
Mask ventilation		0 (0%)	74 (85.06%)	37 (97.37%)	*p* < 0.001 OR = 932.630 (54.497–15960.396)	*p* < 0.001 OR = 4225.000 (168.189–106134.638)	*p* = 0.077 OR = 6.500 (0.819–51.610)
Mech. ventilation		0 (0%)	12 (13.79%)	17 (44.74%)	*p* = 0.022 OR = 27.980 (1.629–480.697)	*p* < 0.001 OR = 137.558 (7.951–2379.895)	*p* < 0.001 OR = 5.060 (2.092–12.239)
Intubation		0 (0%)	12 (13.79%)	18 (47.37%)	*p* = 0.022 OR = 27.980 (1.629–480.697)	*p* < 0.001 OR = 152.512 (8.820–2637.158)	*p* < 0.001 OR = 5.625 (2.330–13.580)
Epinephrin/Adrenalin		0 (0%)	0 (0%)	0 (0%)	*p* = 0.986 OR = 0.966 (0.019–49.230)	*p* = 0.696 OR = 2.195 (0.043–112.683)	*p* = 0.683 OR = 2.273 (0.044–116.660)
Chest compressions		0 (0%)	0 (0%)	1 (2.63%)	*p* = 0.986 OR = 0.966 (0.019–49.230)	*p* = 0.245 OR = 6.760 (0.269–169.815)	*p* = 0.237 OR = 7.000 (0.279–175.802)
NICU interventions							
Surfactant replacement therapy		0 (0%)	38 (43.68%)	32 (84.21%)	*p* < 0.001 OR = 131.444 (7.900–2187.150)	*p* < 0.001 OR = 845.000 (46.272–15431.093)	*p* < 0.001 OR = 6.877 (2.609–18.132)
Caffeine citrate		0 (0%)	83 (95.40%)	38 (100.0%)	*p* < 0.001 OR = 3135.889 (166.212–59164.369)	*p* < 0.001 OR = 13013.000 (253.464–668095.666)	*p* = 0.344 OR = 4.150 (0.218–79.018)
Postnatal steroids		0 (0%)	16 (18.39%)	33 (86.84%)	*p* = 0.011 OR = 39.000 (2.299–661.611)	*p* < 0.001 OR = 1029.364 (55.371–19136.133)	*p* < 0.001 OR = 29.288 (9.889–86.743)
Mech. ventilation		0 (0%)	36 (41.38%)	33 (86.84%)	*p* < 0.001 OR = 119.777 (7.195–1993.876)	*p* < 0.001 OR = 1029.364 (55.371–19136.133)	*p* < 0.001 OR = 9.350 (3.329–26.265)
Mech. ventilation (days)		0	2 (1–21)	19 (1–60)	ND	ND	< 0.001
CPAP		0 (0%)	83 (95.40%)	38 (100.0%)	*p* < 0.001 OR = 3135.889 (166.212–59164.369)	*p*< 0.001 OR = 13013.000 (253.464–668095.666)	*p* = 0.344 OR = 4.150 (0.218–79.018)
Oxygen (days)		0	11.5 (1–78)	85.5 (23–126)	ND	ND	< 0.001
Discharge							
Oxygen at discharge		0 (0%)	1 (1.15%)	8 (21.05%)	*p* = 0.512 OR = 2.931 (0.118–72.959)	*p* = 0.009 OR = 47.098 (2.638–840.797)	*p* = 0.004 OR = 22.933 (2.753–191.066)
Duration of hospital stay (days)		5 (4–10)	54.5 (18–119)	104.0 (46–160)	< 0.001	<0.001	< 0.001
Weight (g)		3200 (2380–4290)	2320 (1170–3680)	2885 (1675–3980)	< 0.001	<0.001	< 0.001

Data are presented as median (range) for continuous variables and as number (percent) for categorical variables. Continuous variables were compared using the Mann-Whitney test and categorical variables were compared using odds ratio test. For the comparison of fetal sex and mode of delivery the Chi-squared test was used.

*p*-value^1^, the comparison among normal term children and preterm children without BPD;

*p*-value^2^, the comparison among normal term children and preterm children with BPD;

*p*-value^3^, the comparison among preterm children w/ and w/o BPD. BPD, bronchopulmonary dysplasia; CS, Cesarean section; MgSO_4_, magnesium sulfate; GDM, gestational diabetes mellitus; PPROM, preterm premature rupture of membranes; FGR, fetal growth restriction; SGA, small for gestational age; RDS, respiratory distress syndrome; PDA, patent ductus arteriosus; NEC, necrotizing enterocolitis; PIVH, periventricular/intraventricular hemorrhage; PVL, periventricular leukomalacia; ROP, retinopathy of prematurity; CPAP, continuous positive airway pressure.

### 2.2 Echocardiographic examination

Comprehensive two-dimensional echocardiographic examination was performed using a Philips EPIQ Elite ultrasound system (Philips Ultrasound, United States) equipped with a phased array transducer (frequency range 2–9 MHz). Morphological and functional parameters were comprehensively assessed. The examination included color flow Doppler, pulsed and continuous wave Doppler modalities with adaptive technology.

Tricuspid valve (TV), TAPSE, pulmonary artery acceleration time (PAAT), and right ventricular ejection time (RVET) were examined to determine PVR. A test for the diagnosis of TV insufficiency (presence and degree of TV regurgitation) was performed (29). TV regurgitation is a common sign of PH. Decreased TAPSE correlates with impaired right ventricular function and may be associated with increased PVR. Age-based TAPSE z-score value < −2 SD (standard deviation) was used as cut-off (30, 31).

A shortened PAAT values indicate increased pulmonary artery pressure (PAP) and potentially increased PVR. Abnormal PAAT values (age- and sex-adjusted z-scores < −1.33 and < −2.0) were predictive of PH (32, 33). To account for heart rate variability, PAAT was adjusted for RVET (PAAT/RVET). Changes in RVET may also reflect changes in pulmonary circulation. PAAT/RVET-indexes < 0.31 and < 0.28 were used as cut-offs, since they represent good predictors of PH (33–35).

All examinations were performed by an expert in pediatric echocardiography. Children with abnormal findings were referred to a pediatric cardiologist.

### 2.3 Examination of MicroRNA profiles

Samples of unclotted peripheral venous blood (200 μL) were processed as previously described (36–41). Homogenized leukocyte lysates were prepared using QIAamp RNA Blood Mini Kit (Qiagen, Germany) and total RNA enriched for small RNAs was extracted using a mirVana microRNA Isolation kit (Ambion, United States). A panel of 29 microRNAs implicated in cardiac and pulmonary development, as well as in the pathogenesis of cardiovascular and pulmonary disorders, was selected based on previously published evidence demonstrating their dysregulated expression across a broad spectrum of cardiovascular diseases. These microRNAs were chosen due to their established roles in the molecular mechanisms underlying disease development and progression. Detailed information regarding their biological functions and associations with specific cardiovascular and pulmonary pathologies has been described in our previous studies (36–41). Particular microRNAs were transcribed into cDNA using microRNA-specific stem-loop RT primers. cDNA was mixed with specific TaqMan MGB primers and probes (TaqMan MicroRNA Assay, Applied Biosystems, United States), and the constituents of the TaqMan Universal PCR Master Mix (Applied Biosystems, United States). Subsequent real-time PCR was performed as previously described (36–41). The expression of studied microRNAs was normalized to geometric mean of RNU58A and RNU38B and comparative Ct method was used to determine the expression of each microRNA (42, 43).

### 2.4 Statistical analysis

The odds ratios (OR), 95% confidence intervals (95% CI), and *p*-values were used to compare the presence of echocardiographic findings between groups. The significance level was established at *p* < 0.05.

Age-, sex-, and BMI- adjusted microRNA profiles were compared between groups using Quade’s non-parametric analysis of covariance (ANCOVA) (IBM SPSS 29.0.2.0, Inc., United States). Post-hoc Benjamini-Hochberg (B-H) correction was applied (44). Statistically significant results are marked * for α = 0.05, ** for α = 0.01 and *** for α = 0.001.

Box plots were generated in case of different microRNA profiles between the groups (MedCalc Software bvba, Belgium). In addition, ROC (receiver operating characteristic) curves were processed (MedCalc Software bvba, Belgium).

Factor analysis (FA) was performed on the entire set of microRNAs under study (29 variables) using Principal Component Analysis (PCA) with varimax rotation. The suitability of the data for FA was confirmed using the Kaiser-Meyer-Olkin (KMO) test and Bartlett’s test of sphericity. KMO values between 0.80 and 0.90 are values achieving a great level of acceptability. The significant result of Bartlett’s test of sphericity (*p* < 0.05) confirms that the data are suitable for FA. Three-dimensional scatter plots (IBM SPSS, Inc., United States) were used to visualize the variance between groups.

Correlations between variables including relative microRNA gene expression and Doppler parameters (TAPSE age-based z-score values, PAAT age- and sex-adjusted z-score values, and PAAT/RVET ratio values) were calculated using the Spearman’s rank correlation coefficient (ρ). The correlation coefficient values within < −0.5; 0 > indicate week negative correlations. *P*-values of *p* < 0.05 were considered significant.

## 3 Results

### 3.1 Echocardiographic findings in normal term and preterm children

Seventeen of 92 normal term children (18.48%) had abnormal echocardiographic findings. The most common abnormal echocardiographic findings were heart valve regurgitations (*n* = 9) or patent foramen ovale (PFO, *n* = 5). The presence of the most common congenital heart defects (*n* = 2) and arrhythmia (*n* = 1) was found less frequently.

Preterm children exhibited a broader spectrum of echocardiographic abnormalities compared with normal term children. In addition to valvular regurgitation (*n* = 15), PFO (*n* = 11), and their combination (*n* = 5), several other cardiac abnormalities were identified exclusively in the preterm group, including patent ductus arteriosus (PDA) associated with PFO (*n* = 2), coronary artery fistula (*n* = 1), ventricular septal defect (VSD) requiring pulmonary artery banding (*n* = 1), and echocardiographic signs suggestive of increased PVR (*n* = 21). The most severe clinical presentations were characterized by the coexistence of structural cardiac abnormalities and elevated PVR (*n* = 12), indicating a more pronounced cardiopulmonary burden in these individuals.

Overall, a higher incidence of abnormal echocardiographic findings was detected in preterm children regardless of the previous BPD occurrence (46.58%) compared to normal term children (18.48%) (Table 2). Expanded information on abnormal echocardiographic findings in children is provided in Supplementary Table 1.

**TABLE 2:** The incidence of abnormal echocardiographic findings in preterm children.

Echocardiographic findings	Normal term (*n* = 92)	No BPD preterm (*n* = 101)	BPD preterm (*n* = 45)	*p*^1^-value OR (95% CI)	*p*^2^-value OR 95% CI	*p*^3^-value OR 95% CI
Only increased PVR	0/92 (0%)	15 (14.85%)	6 (13.33%)	*p* = 0.015 OR = 33.150 (1.954–562.550)	*p* = 0.021 OR = 30.443 (1.674–553.563)	*p* = 0.809 OR = 0.882 (0.318–2.445)
Other abnormal echocardiographic findings	17/92 (18.48%)	23 (22.77%)	12 (26.67%)	*p* = 0.463 OR = 1.301 (0.644–2.626)	*p* = 0.273 OR = 1.604 (0.689–3.734)	*p* = 0.611 OR = 1.233 (0.550–2.767)
Increased PVR and other abnormal echocardiographic findings	0/92 (0%)	8 (7.92%)	4 (8.89%)	*p* = 0.054 OR = 16.818 (0.957–295.635)	*p* = 0.046 OR = 20.060 (1.056–381.227)	*p* = 0.844 OR = 1.134 (0.323–3.979)
Increased PVR and/or other abnormal echocardiographic findings overall	17/92 (18.48%)	46/101 (45.54%)	22/45 (48.89%)	*p* < 0.001 OR = 3.690 (1.914–7.112)	*p* < 0.001 OR = 4.220 (1.922–9.267)	*p* = 0.708 OR = 1.144 (0.566–2.311)

*p*^1^-value, no BPD preterm group vs. normal term group;

*p*^2^-value, BPD preterm group vs. normal term group;

*p*^3^-value, BPD preterm group vs. no BPD preterm group; PVR, pulmonary vascular resistance.

### 3.2 MicroRNA profiles in preterm children with regard to the previous BPD occurrence

**FIGURE 2:**
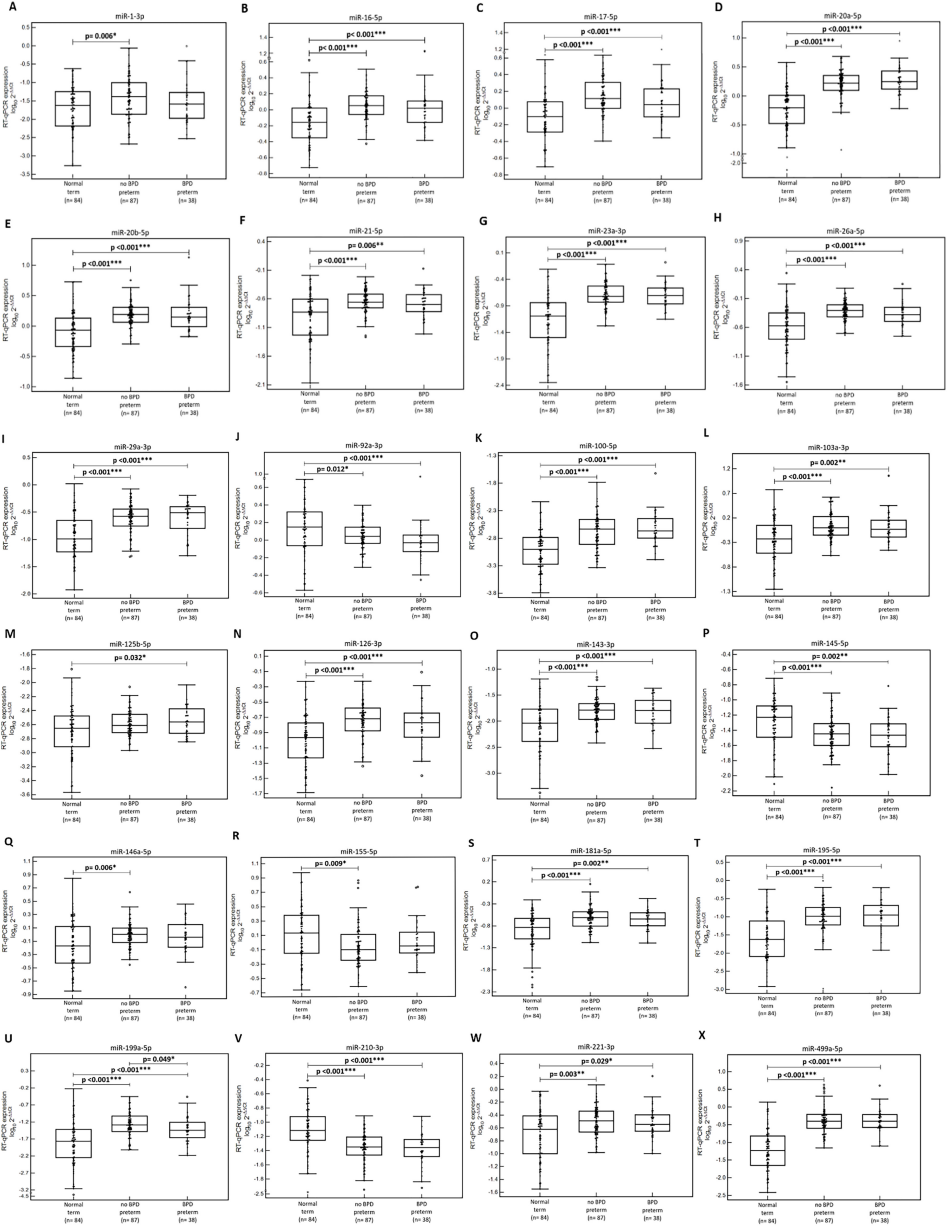
MicroRNA gene expression profiles in preterm children with regard to the previous occurrence of BPD. Only those microRNAs with altered expression are presented **(A–X)**. Age-, sex-, and BMI—adjusted microRNA gene expression profiles were compared between groups using Quade’s non-parametric ANCOVA. *Post-hoc* Benjamini-Hochberg correction was applied. Statistical significant results are marked * for α = 0.05, ** for α = 0.01 and *** for α = 0.001.

Children born preterm shared altered expression of a significant number of microRNAs (20 in total), regardless of a history of BPD, when compared with normal term controls. Among these, 17 microRNAs were upregulated and 3 were downregulated (Figure 2). In addition to these, three other microRNAs with altered expression were identified exclusively in preterm children without BPD: miR-155-5p was downregulated, while miR-1-3p and miR-146a-5p were upregulated (Figure 2). Moreover, upregulation of miR-125b-5p was observed specifically in preterm children diagnosed with BPD (Figure 2). Differences in microRNA expression were also observed between subgroups of preterm children. Notably, miR-199a-5p was upregulated in children without BPD compared to those with BPD (Figure 2).

Gene expression of miR-20a-5p and miR-499a-5p best differentiated between preterm children regardless of the previous BPD occurrence and normal term children (Table 3 and Supplementary Figures 1, 2).

**TABLE 3:** ROC curve parameters—the differentiation between preterm children diagnosed with and without BPD and normal term children (only statistically significant results are presented).

microRNA	BPD preterm		No BPD Preterm	
	AUC 95% CI *p*-value	Sensitivity at 10.0% FPR 95% CI criterion	AUC 95% CI *p*-value	Sensitivity at 10.0% FPR 95% CI criterion
miR-1-3p	NS	NS	0.632 0.555–0.704 0.002	19.54% 8.05–31.03 > 0.117342
miR-16-5p	0.702 0.612–0.781 < 0.001	13.16% 2.17–47.37 > 1.448439	0.770 0.700–0.831 < 0.001	27.59% 4.60–49.43 > 1.449563
miR-17-5p	0.690 0.600–0.771 < 0.001	31.58% 7.89–57.89 > 1.533926	0.769 0.699–0.830 < 0.001	33.33% 5.75–49.43 > 1.545085
miR-20a-5p	0.906 0.840–0.951 < 0.001	65.79% 34.21–81.58 > 1.434205	0.873 0.813–0.919 < 0.001	65.52% 48.28–85.06 > 1.434205
miR-20b-5p	0.746 0.659–0.820 < 0.001	28.95% 10.53–51.80 > 1.814570	0.783 0.713–0.842 < 0.001	34.48% 10.34–54.02 > 1.814570
miR-21-5p	0.658 0.566–0.741 0.001	10.53% 0.00–34.21 > 0.367628	0.692 0.617–0.760 < 0.001	13.79% 1.15–31.03 > 0.369272
miR-23a-3p	0.801 0.719–0.868 < 0.001	28.95% 0.00–55.26 > 0.269989	0.800 0.733–0.858 < 0.001	32.18% 5.75–55.17 > 0.263779
miR-24-3p	NS	NS	NS	NS
miR-26a-5p	0.705 0.616–0.784 < 0.001	13.16% 0.00–39.47 > 0.650859	0.755 0.684–0.817 < 0.001	20.69% 1.15–42.53 > 0.648597
miR-29a-3p	0.756 0.670–0.829 < 0.001	42.11% 15.79–70.34 > 0.349845	0.748 0.676–0.811 < 0.001	26.44% 12.64–57.42 > 0.349845
miR-92a-3p	0.691 0.601–0.772 < 0.001	7.89% 0.00–28.95 ≤ 0.553384	0.621 0.544–0.694 0.007	3.45% 0.00–25.29 ≤ 0.574114
miR-100-5p	0.805 0.723–0.871 < 0.001	42.11% 23.68–65.79 > 0.002490	0.761 0.690–0.823 < 0.001	43.68% 24.14–62.07 > 0.002496
miR-103a-3p	0.679 0.588–0.760 < 0.001	18.42% 5.26–36.84 > 1.723711	0.707 0.633–0.774 < 0.001	24.14% 10.34–35.63 > 1.745513
miR-125b-5p	0.595 0.518–0.669 0.031	10.34% 0.00–20.69 > 0.004567	NS	NS
miR-126-3p	0.690 0.600–0.771 < 0.001	18.42% 0.034–36.84 > 0.248400	0.747 0.675–0.811 < 0.001	26.44% 6.90–45.98 > 0.247654
miR-130b-3p	NS	NS	NS	NS
miR-133a-3p	NS	NS	NS	NS
miR-143-3p	0.676 0.585–0.758 < 0.001	18.42% 2.63–45.16 > 0.032434	0.696 0.621–0.763 < 0.001	8.05% 2.24–27.59 > 0.032681
miR-145-5p	0.690 0.600–0.771 < 0.001	13.16% 2.27–36.84 ≤ 0.017209	0.675 0.599–0.745 < 0.001	16.09% 1.15–38.15 ≤ 0.018607
miR-146a-5p	NS	NS	0.634 0.557–0.706 0.003	5.75% 2.30–20.46 > 1.977922
miR-155-5p	NS	NS	0.630 0.553–0.702 0.003	6.90% 2.30–36.27 ≤ 0.385638
miR-181a-5p	0.690 0.600–0.770 < 0.001	15.79% 2.63–47.37 > 0.353428	0.717 0.643–0.783 < 0.001	19.54% 9.20–45.40 > 0.354006
miR-195-5p	0.799 0.717–0.866 < 0.001	28.95% 5.26–52.63 > 0.160757	0.788 0.719–0.846 < 0.001	29.89% 6.90–47.13 > 0.161941
miR-199a-5p	0.704 0.614–0.783 < 0.001	7.89% 0.00–36.84 > 0.101146	0.771 0.700–0.831 < 0.001	21.84% 9.20–47.13 > 0.101211
miR-210-3p	0.771 0.686–0.842 < 0.001	23.68% 5.26–52.63 ≤ 0.031511	0.746 0.674–0.810 < 0.001	22.99% 9.20–58.28 ≤ 0.031569
miR-221-3p	0.627 0.535–0.713 0.011	7.89% 0.00–31.58 > 0.623438	NS	NS
miR-342-3p	NS	NS	NS	NS
miR-499a-5p	0.893 0.825–0.942 < 0.001	60.53% 10.53–94.74 > 0.331725	0.883 0.825–0.927 < 0.001	59.77% 17.24–83.91 > 0.333789
miR-574-3p	NS	NS	NS	NS

FPR, false positive rate; BPD, bronchopulmonary dysplasia; AUC, area under the ROC (receiver operating characteristic) curve; CI, confidence interval; NS, non-significant results.

### 3.3 MicroRNA profiles in preterm children with regard to current echocardiographic findings

Based on the echocardiographic examination, children in each group (preterm and term) were further subdivided into subgroups according to the presence of functional or morphological abnormalities of the heart/valves (no findings = 0, abnormal findings = 1) and the presence of signs of pulmonary vascular resistance (no PVR = 0, with PVR = 1) (Table 4).

**TABLE 4:** Study subgroups according to current echocardiographic findings and PVR presence.

Stugy groups	Current echocardiographic findings	Presence of PVR	Subgroup ID	Number of subjects
Normal term	No findings	No PVR	**1**	72
	Abnormal findings	No PVR	**2**	12
Preterm	No findings	No PVR	**3**	63
	No findings	With PVR	**4**	18
	Abnormal findings	No PVR	**5**	30
	Abnormal findings	With PVR	**6**	9

PVR, pulmonary vascular resistance.

Expression of multiple microRNAs differed significantly among the studied subgroups; however, the differences were solely dependent on the term of birth (preterm vs. normal term) (subgroups 3, 4, 5, 6 vs. subgroups 1 or 2), regardless of the presence of functional or morphological abnormalities of the heart/valves and/or the presence of PVR. MicroRNA profiles did not differ between equivalent subgroups of children with respect to the presence of functional or morphological abnormalities of the heart/valves (subgroups 1 vs. 2, subgroups 3 vs. 5, and subgroups 4 vs. 6), the presence of PVR (subgroups 3 vs. 4, and subgroups 5 vs. 6), and their combination (subgroups 3 vs. 6) (Supplementary Figure 3). Only a weak negative correlation between miR-143-3p and PAAT z-score (−0.237, *p* = 0.009) was detected (Supplementary Figure 4).

### 3.4 PCA results

**FIGURE 3:**
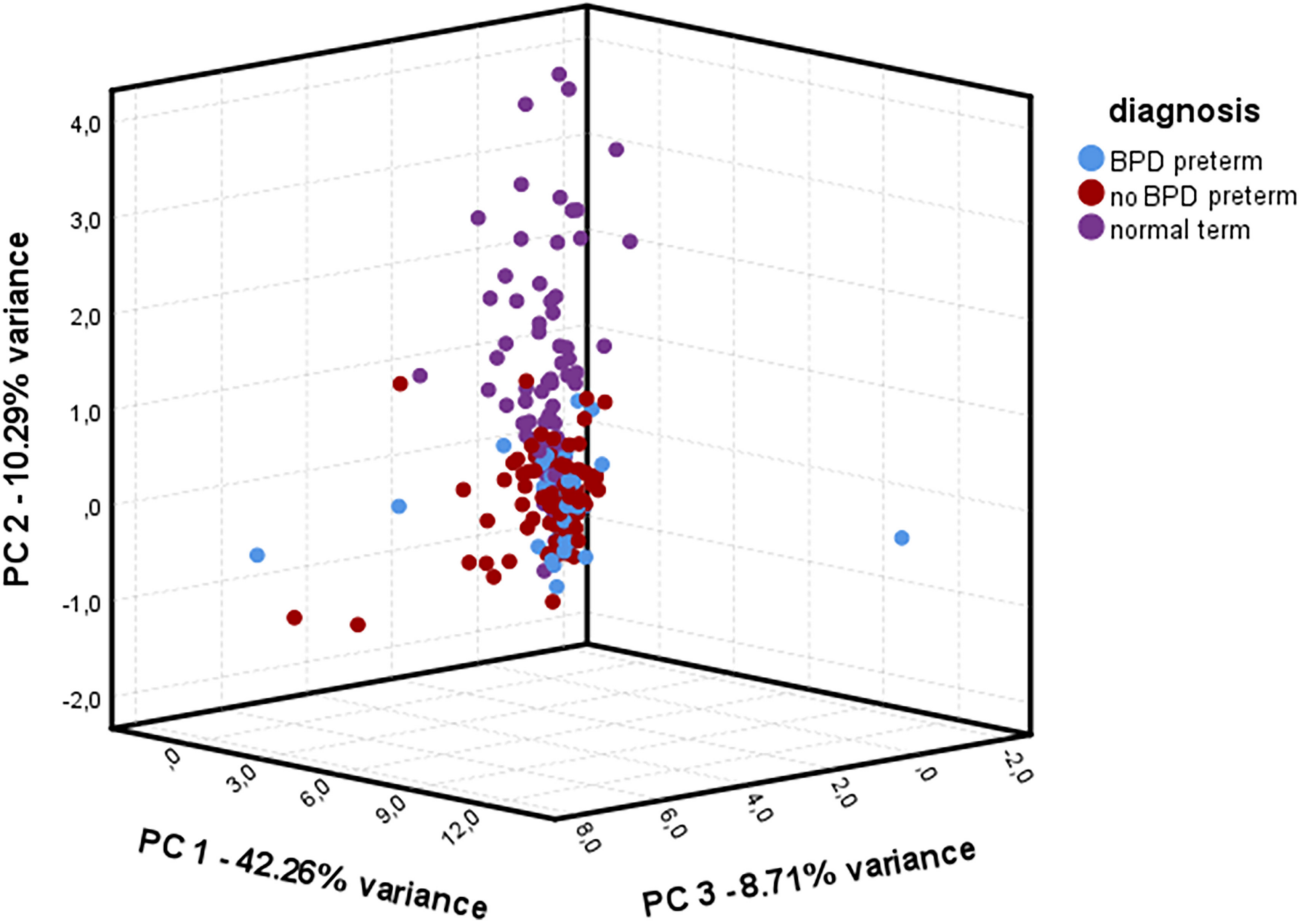
Principal component analysis (PCA).Three-dimensional PCA plot demonstrates a clear distinction between normal term children and preterm children. In contrast, preterm children with a history of BPD and those without BPD show considerable overlap, with no evident separation between the two groups.

There was a clear distinction between preterm children and normal term children. Regarding the previous BPD occurrence, no distinction between the clusters was apparent, because preterm children had very similar microRNA profiles (Figure 3).

The validity of the FA was confirmed using the KMO test (0.886) and Bartlett’s test of sphericity (χ^2^ = 5857.55, df = 406, *p* < 0.001). The cumulative variance using the first six principal components (PCs) that had initial eigenvalues greater than 1 was 75.58%. The first six PCs explained 42.26, 10.29, 8.71, 5.89, 4.28, and 4.15% of the variance, respectively. The number of variables with large positive loadings ( > 0.500) or large negative loadings ( < −0.500) in each PC reached 6 microRNAs (PC1), 7 microRNAs (PC2), 6 microRNAs (PC3), 5 microRNAs (PC4), 4 microRNAs (PC5), and 3 microRNAs (PC6). The first three PCs explained 61.26% of the variance between groups (Table 5).

**TABLE 5:** Results of factor analysis.

Factor analysis, total variance	Principal component*					
	PC 1	PC 2	PC 3	PC 4	PC 5	PC 6
Explained variance (%)	42.26%	10.29%	8.71%	5.89%	4.28%	4.15%
Cumulative variance explained (%)	42.26%	52.55%	61.26%	67.15%	71.43%	75.58%
Number of variables with loadings > 0.500	6	7	6	5	4	3
miR-1-3p	0.199	−0.019	**0.906**	0.136	0.041	0.015
miR-16-5p	**0.937**	0.113	0.124	0.171	0.097	0.013
miR-17-5p	**0.812**	0.123	0.153	0.470	0.075	−0.026
miR-20a-5p	**0.544**	−0.011	0.171	0.307	0.209	**0.516**
miR-20b-5p	**0.812**	0.189	0.133	0.183	0.286	0.238
miR-21-5p	0.246	0.164	**0.518**	0.295	0.399	0.277
miR-23a-3p	0.148	0.129	0.368	**0.691**	0.194	0.310
miR-24-3p	0.264	**0.679**	0.310	0.207	0.380	0.193
miR-26a-5p	0.333	0.313	0.442	0.422	0.432	0.214
miR-29a-3p	0.056	0.092	0.340	0.087	**0.629**	0.442
miR-92a-3p	**0.662**	**0.662**	0.049	0.065	−0.106	−0.068
miR-100-5p	0.112	−0.052	0.063	−0.088	**0.686**	0.305
miR-103a-3p	**0.688**	0.180	0.150	**0.580**	0.191	−0.053
miR-125b-5p	0.135	0.238	0.087	0.101	**0.755**	0.032
miR-126-3p	0.494	0.204	**0.520**	0.347	0.320	0.275
miR-130b-3p	0.143	**0.600**	0.105	0.249	0.270	0.204
miR-133a-3p	0.007	0.222	**0.849**	0.062	0.046	0.014
miR-143-3p	0.039	0.335	0.468	0.233	0.314	0.475
miR-145-5p	−0.015	**0.729**	**0.516**	−0.025	0.041	0.100
miR-146a-5p	0.154	0.294	0.192	0.175	**0.723**	−0.120
miR-155-5p	−0.020	0.365	−0.050	0.199	0.472	−**0.541**
miR-181a-5p	0.195	0.065	**0.612**	0.175	0.315	0.196
miR-195-5p	0.026	0.111	0.079	0.175	0.173	**0.717**
miR-199a-5p	0.199	0.021	0.149	**0.724**	0.024	0.045
miR-210-3p	0.123	**0.754**	−0.016	−0.159	−0.003	−0.067
miR-221-3p	0.402	0.349	0.225	0.468	0.162	0.373
miR-342-3p	0.058	**0.740**	0.057	0.105	0.433	−0.031
miR-499a-5p	0.382	−0.063	0.004	**0.832**	0.020	0.057
miR-574-3p	0.239	**0.617**	0.187	**0.528**	0.194	0.168

*Weights from rotated components with Varimax method and Kaiser Normalization. The variables with large positive loadings (>0.500) or large negative loadings (<0.500) are marked in bold.

Following the additional inclusion of gestational birth weight in the analysis, the PCA yielded results comparable to those obtained previously using only microRNA biomarkers. Sampling adequacy remained excellent, as demonstrated by the KMO measure of 0.885, while Bartlett’s test of sphericity confirmed the suitability of the data for PCA (χ^2^ = 6079.24, df = 435, *p* < 0.001). The first six PCs accounted for 75.29% of the total variance, with individual contributions of 41.03, 11.04, 9.10, 5.94, 4.15, and 4.03%, respectively. Notably, the first three PCs collectively explained 61.17% of the variance, indicating that the addition of gestational birth weight did not substantially alter the overall dimensional structure and discriminatory capacity of the model.

To further quantify its effect, correlation analysis was performed between gestational birth weight and the principal component scores derived from the original PCA based on microRNA expression data. Gestational birth weight showed a weak but statistically significant correlation with PC1 (*r* = −0.243, *p* < 0.001), indicating that it is not a major contributor to the primary source of variance captured by the PCA. In contrast, no correlation was observed with PC2 (*r* = −0.076, *p* = 0.276), suggesting that birth weight does not contribute to secondary variability in the dataset.

## 4 Discussion

Our current study focused on preschool and school-age children born preterm due to the occurrence of severe pregnancy-related complications, which required in most cases termination of pregnancy by cesarean section (CS). We evaluated the associations between the microRNA profiles in peripheral venous blood and the previous BPD occurrence and the concomitant occurrence of abnormal echocardiographic findings, including an increased PVR.

Like other studies focusing on preschool and school-age children, we detected an overall increased incidence of abnormal echocardiographic findings in preterm children compared to normal term children. Severe morphological findings such as coronary artery fistula, surgery for VSD with PAB (pulmonary artery banding), quadricuspid aortic valve, and PDA were present only in preterm children, regardless of the previous BPD occurrence. Increased PVR was detected only in preterm children, also regardless of the previous BPD occurrence (26). PH was not detected in preterm children (25).

The current study showed that preschool and school-age preterm children shared largely altered microRNA profiles regardless of the previous BPD occurrence (20 microRNAs in total, of which 17 were upregulated and 3 downregulated) compared to normal term children. Additional 3 microRNAs in preterm children without BPD and 1 microRNA in preterm children with BPD differed in expression profiles from normal term children. Only dysregulation of miR-199a-5p differentiated within preterm children with and without BPD.

The present study also demonstrated that microRNA profiles did not differ between groups of preterm children or groups of normal term children with respect to the actual presence of abnormal echocardiographic findings. The microRNA profiles did not differ between preterm children who did or did not have a detected increased PVR as a single finding. In the presence of other abnormal echocardiographic findings (functional or morphological abnormalities of the heart/valves), there was also no abnormal expression of any microRNA in children with an increased PVR.

The observation of only a single relationship (a weak negative correlation between miR-143-3p and PAAT z-score) between PVR markers and the studied microRNAs supports the interpretation of a limited association between microRNA profiles and increased PVR in this cohort.

The results of this study largely confirmed the results of our previous study, although these studies were only partially consistent in design. Our previous study focused on studying the microRNA profiles in preschool and school-age children born before 37 gestational weeks due to the onset of preterm prelabor rupture of membranes (PPROM) or spontaneous preterm birth (PTB) without the occurrence of other pregnancy-related complications (45). This pilot study was designed to compare the microRNA profiles between children with normal and abnormal clinical findings, where the children were either overweight/obese, had prehypertension/hypertension or abnormal echocardiographic findings. We newly evaluated the associations between the microRNA profiles and the previous BPD occurrence and the concomitant occurrence of abnormal echocardiographic findings, including an increased PVR. A total of 17 microRNAs showed similar profiles in both studies in preterm children, regardless of gestational age at delivery. However, preterm children also showed aberrant expression of other microRNAs after rigorous statistical evaluation of the data including adjustment for age, sex and BMI and B-H correction, that were not yet dysregulated in preterm children born in later gestational weeks due to the occurrence PPROM or PTB only (miR-92a-3p, miR-145-5p, miR-155-5p, and miR-210-3p) (45).

Several studies have shown dysregulation of microRNAs in whole peripheral blood of preterm neonates, but mostly only in the early period of life ranging from 3 days to 3 months (46–48), but not later in life. Guler et al. (47) demonstrated an upregulation of microRNAs (miR-17-5p, miR-20a-5p, miR-21-5p, miR-23a-3p, miR-126-3p, and miR-145-5p) in premature neonates with BPD and suggestive PH (49). In the present study, we evaluated the expression of these microRNAs in preschool and school-age preterm children. However, in our study, all these microRNAs were dysregulated regardless of the previous BPD occurrence and current evidence of an increased PVR. Guler et al. (47) also reported upregulation of miR-221-3p in premature infants with BPD regardless of suggestive PH in their early life. However, we again observed upregulation of miR-221-3p in preschool and school-age preterm children regardless of the prior BPD occurrence and the concomitant evidence of an increased PVR. Furthermore, Gong et al. (48) reported downregulation of miR-574-3p in premature neonates with BPD in their early life, that was not dysregulated in our study.

We believe that microRNA dysregulation detected at an early age, as shown in previous studies, may persist, intensify, or gradually normalize with age, depending on the specific microRNA and underlying disease. However, it is also possible that changes in microRNA levels may also be explained by differences in study design, timing of sampling, or analytical methods.

In addition, detailed microRNA profiling was performed to classify patients according to disease subtypes (BPD-associated PH, PH associated with congenital diaphragmatic hernia/pulmonary hypoplasia or congenital heart disease, and idiopathic/hereditary PH), to correlate microRNA profiles with disease severity, and to monitor response to treatment in school-age children with PH (50). Circulating plasma microRNA levels were subject of interest. In the set of 983 microRNAs profiled, none was identified as associated with PH caused by BPD (50), which is consistent with the results of our study, where no microRNA had been conclusively identified to be dysregulated in the presence of increased PVR, a risk factor for PH development.

## 5 Conclusion

Although the study included relatively small groups of children, our findings confirmed a higher prevalence of echocardiographic abnormalities in preterm preschool and school-age children with a birth weight < 1,500 g and/or gestational age at birth < 32 weeks compared to term-born peers. However, it should be noted that no relationships were observed between abnormal echocardiographic findings and microRNA levels in peripheral venous blood in children born at comparable gestational age regardless of birth history.

Nevertheless, the effect of preterm birth on microRNA profiles is evident, as reported in other studies (46–48) and also in our study. Whether these epigenetic modifications induced by prematurity will either normalized or progressively exacerbates with age and whether would be predictive of later healthy complications in adulthood remains a subject for further investigation.

## Data Availability

The raw data supporting the conclusions of this article will be made available by the authors, without undue reservation.
