# Supplementary material for: MicroRNA profiles in preterm children with regard to BPD and echocardiographic findings
Source: Front Med (Lausanne). 2026 Aug 4;13:1913675. doi: 10.3389/fmed.2026.1913675 (PMC13481854; doi:10.3389/fmed.2026.1913675)
Supplement: Supplementary file 1 [file Supplementary_file_1.docx]

Supplementary Material

**Supplementary Table 1.** Expanded information on abnormal echocardiographic findings in preterm children

|  | Normal term  (n=92) | No BPD preterm  (n=101) | BPD preterm  (n=45) |
| --- | --- | --- | --- |
| Only increased PVR | 0/92 (0 %) | 15 (14.85 %)  ↓PAAT (n=9)  ↓PAAT + ↓PAAT/RVET (n=5)  ↓PAAT + ↓TAPSE (n=1) | 6 (13.33 %)  ↓PAAT (n=4)  ↓PAAT + ↓PAAT/RVET (n=2) |
| Other echocardiographic findings | 17/92 (18.48 %)  heart valve regurgitation (n=9)  PFO (n=5)  VSD (n=1)  bicuspid aortic valve (n=1)  arrhythmia (n=1) | 23 (22.77 %)  heart valve regurgitation (n=12)  PFO (n=6)  PFO + heart valve regurgitation (n=3)  PDA + PFO (n=1)  coronary artery fistula (n=1) | 12 (26.67 %)  heart valve regurgitation (n=3)  PFO (n=5)  PFO + heart valve regurgitation (n=2)  PDA + PFO (n=1)  condition after surgical repair of VSD with PAB (n=1) |
| Increased PVR and other echocardiographic findings | 0/92 (0 %) | 8 (7.92 %)  heart valve regurgitation (n=3)  PFO (n=3)  PFO + heart valve regurgitation (n=1)  quadricuspid aortic valve (n=1)  ↓PAAT (n=3)  ↓PAAT + ↓PAAT/RVET (n=3)  ↓PAAT + ↓TAPSE (n=2) | 4 (8.89 %)  heart valve regurgitation (n=4)  ↓PAAT (n=1)  ↓PAAT + ↓PAAT/RVET (n=3) |
| Increased PVR and/or other abnormal echocardiographic findings overall | 17/92 (18.48 %) | 46/101(45.54 %) | 22/45 (48.89 %) |

BPD, bronchopulmonary dysplasia; PVR, pulmonary vascular resistance; PAAT, pulmonary artery acceleration time; RVET, right ventricular ejection time; TAPSE, tricuspid annular plane systolic excursion; PFO, patent foramen ovale; PDA, patent ductus arteriosus; VSD, ventricular septal defect; PAB, pulmonary artery banding

**Supplementary Figure 1.**

**Supplementary Figure 1.** ROC curves showing the differentiation between preterm children with BPD and normal term children.

**Supplementary Figure 2.**

**Supplemetary Figure 2.** ROC curves showing the differentiation between preterm children without BPD and normal term children.

**Supplemetary Figure 3.**

**Supplementary Figure 3.** MicroRNA gene expression profiles in the groups of normal term children and preterm children with regard to current echocardiographic findings. Age-, sex-, and BMI- adjusted microRNA gene expression profiles were compared between groups using Quade's non-parametric ANCOVA. Post-hoc Benjamini-Hochberg correction was applied. Statistical significant results are marked * for α=0.05, ** for α=0.01 and *** for α=0.001.

**Supplementary Figure 4.**

**Supplementary Figure 4.** Correlation between miR-143-3p and PAAT z-score. Correlation between variables was calculated using Spearman’s rank correlation coefficient. The correlation coefficient within the range <-0.5; 0> with a p-value p < 0.05 indicates a week negative correlation.
